# Effect of praziquantel treatment of *Schistosoma mansoni *during pregnancy on intensity of infection and antibody responses to schistosome antigens: results of a randomised, placebo-controlled trial

**DOI:** 10.1186/1471-2334-9-32

**Published:** 2009-03-18

**Authors:** Robert Tweyongyere, Patrice A Mawa, Nicholas O Emojong, Harriet Mpairwe, Frances M Jones, Trinh Duong, David W Dunne, Birgitte J Vennervald, Eli Katunguka-Rwakishaya, Alison M Elliott

**Affiliations:** 1 Faculty of Veterinary Medicine, Makerere University, Kampala, Uganda; 2 Medical Research Council/Uganda Virus Research Institute-Uganda Research Unit on AIDS, Kampala, Uganda; 3 Department of Pathology, University of Cambridge, Cambridge, UK; 4 London School of Hygiene and Tropical Medicine, London, UK; 5 DBL-Centre for Health Research and Development, Department of Disease Biology, University of Copenhagen, Copenhagen, Denmark

## Abstract

**Background:**

Praziquantel treatment of schistosomiasis during pregnancy was only recommended in 2002; hence the effects of treatment during pregnancy are not fully known. We have therefore evaluated the effects on infection intensity and the immunological effects of praziquantel treatment against *Schistosoma mansoni *during pregnancy, compared with treatment after delivery.

**Methods:**

A nested cohort of 387 *Schistosoma mansoni *infected women was recruited within a larger trial of de-worming during pregnancy. Women were randomised to receive praziquantel or placebo during pregnancy. All women were treated after delivery. Infection intensity after treatment was assessed by a single Kato-Katz examination of stool samples with duplicate slides and categorised as undetected, light (1–99 eggs per gram (epg)), moderate (100–399 epg) or heavy (≥400 epg). Antibodies against *S. mansoni *worm and egg antigens were measured by ELISA. Results were compared between women first treated during pregnancy and women first treated after delivery.

**Results:**

At enrolment, 252 (65.1%) of the women had light infection (median (IQR) epg: 35 (11, 59)), 75 (19.3%) moderate (median (IQR) epg: 179(131, 227)) and 60 (15.5%) had heavy infection (median (IQR) epg: 749 (521, 1169)) with *S. mansoni*. At six weeks after praziquantel treatment during pregnancy *S. mansoni *infection was not detectable in 81.9% of the women and prevalence and intensity had decreased to 11.8% light, 4.7% moderate and 1.6% heavy a similar reduction when compared with those first treated after delivery (undetected (88.5%), light (10.6%), moderate (0.9%) and heavy (0%), p = 0.16). Parasite specific antibody levels were lower during pregnancy than after delivery. Praziquantel treatment during pregnancy boosted anti-worm IgG isotypes and to a lesser extent IgE, but these boosts were less pronounced than in women whose treatment was delayed until after delivery. Praziquantel had limited effects on antibodies against egg antigens.

**Conclusion:**

*S mansoni *antigen-specific antibody levels and praziquantel-induced boosts in antibody levels were broadly suppressed during pregnancy, but this was not associated with major reduction in the efficacy of praziquantel. Long-term implications of these findings in relation to resistance to re-infection remain to be explored.

**Trial registration:**

International Standard Randomised Controlled Trial Number for the current study: ISRCTN32849447 http://www.controlled-trials.com/ISRCTN32849447/elliott

## Background

Praziquantel treatment of human schistosomiasis during pregnancy and lactation was avoided [1] from the time it became available, in 1979, until an informal consultation by the World Health Organisation in 2002. It was then recommended that pregnant and lactating women with schistosomiasis should be treated [2,3]. This recommendation was based on animal studies, as well as case reports of inadvertent or necessary treatment of pregnant women, which showed no evidence of adverse effects. However, since the benefits and risks of treatment during pregnancy had not been studied, a WHO scientific working group in 2005 called for randomised, placebo-controlled trials of treatment during pregnancy for all species of human schistosomes in both low and high transmission areas [4]. We here report findings from the first such trial (Elliott et al., 2007). In particular, we describe the results of a sub-study designed to examine the immunological effects of treating *Schistosoma mansoni *with praziquantel during pregnancy, compared with the effects of treatment after delivery.

Praziquantel is the drug of choice against all schistosome infections and has shown reliable therapeutic effectiveness. Regular treatment of populations in endemic areas alleviates severe morbidity [5]. One factor that may influence the efficacy of praziquantel is the immune status of the host. Studies have demonstrated that the mode of action of praziquantel involves unique synergy with the host immune responses: praziquantel-induced damage of surface membranes of schistosomes [6-8] exposes the antigens for immune attack [9,10] and, in particular, there is evidence that the efficacy of praziquantel against *S. mansoni *is to some extent dependent on antibodies [11-14]. At the same time, praziquantel treatment of *S. mansoni *causes a boost in parasite-specific antibody responses [15] and there is evidence that some boosts in antibody levels, particularly in immunoglobulin (Ig)E production, may be related to resistance to re-infection [16,17]. However, immune responses are normally altered during pregnancy [18] to allow foetal allograft retention [19-22] and it is therefore of concern that praziquantel treatment during pregnancy may be less effective than treatment in non-pregnant women. For this reason, within our study of the effect of praziquantel during pregnancy on immune responses to schistosome antigens, we have also examined the effects of praziquantel on the intensity of *S. mansoni *infection and have compared effects of treatment during pregnancy with effects of treatment after delivery. We have previously reported that schistosome antigen-specific cytokine responses were suppressed during pregnancy and that boosts in cytokine responses after praziquantel treatment were smaller during pregnancy than after delivery, in a sub-group for whom data on cytokine responses was available [23]. We here report effects of praziquantel treatment during pregnancy on *S. mansoni *intensity and on anti-schistosome antibody responses.

## Methods

### Study design

A nested cohort of 387 pregnant women having schistosomiasis mansoni was enrolled within a larger mother and baby cohort study on "the impact of helminths on the response to immunization and on susceptibility to infectious diseases in childhood in Uganda" (ISRCTN 32849447; http://www.controlled-trials.com/ISRCTN32849447/elliott) [24]. The study was a randomised, double-blind placebo-controlled trial using praziquantel versus placebo and albendazole versus placebo during pregnancy in a 2 × 2 factorial design. Details of the larger cohort and nested cohort have been described [23,24]. Briefly: consenting mothers gave blood for investigations including malaria parasitology, HIV serology and immunological assays, and were asked to provide stool samples for examination for helminth ova. After submission of the first stool sample, they were randomised to receive a single dose of either praziquantel (40 mg/kg), or placebo and albendazole (400 mg) or placebo. At six weeks after delivery all mothers received praziquantel and albendazole treatment. Follow-up samples were obtained at six weeks post-enrolment (while still pregnant), six weeks after delivery (before post-delivery treatment) and twelve weeks after delivery (six weeks after post-delivery treatment). Figure one is a schematic presentation of the follow-up time points, the respective interventions, and samples collected.

Recruitment of the larger cohort was between April 2003 and November 2005, but recruitment to the nested cohort, which constituted women who had *S. mansoni *ova in stool, and which included additional, six-week post-treatment follow up visits, began in November 2003.

### Ethical consideration

Informed consent was obtained from each participant to enrol in the study and provide samples as described previously [24]. Ethical clearance for the study was obtained from the Science and Ethics Committee of Uganda Virus Research Institute – Ministry of Health, the Uganda National Council for Science and Technology and the London School of Hygiene and Tropical Medicine.

### Parasitological diagnosis

A single stool sample per individual was examined for *S. mansoni *ova and other helminth ova including hookworm by duplicate Kato-Katz thick smears [25] at respective time points. *S. mansoni *infection intensity was expressed in eggs per gram (epg) of stool and categorised as light (1–99 epg), moderate (100–399 epg) or heavy (≥400 epg).

### Parasite antigens

Antigen extracts of *S. mansoni *adult worm (SWA) and egg (SEA) were prepared as previously described [10,26].

### Assay of antibodies against schistosome

Immunoglobulins (Ig) G1, G2, G3, G4, E and M against SWA and IgG1, IgG2, IgG3, IgG4 and IgE against SEA were measured in the plasma of the subjects in duplicates per sample using an ELISA method as described elsewhere [27,28]. Briefly: flat bottom 96-well styrene microtitre -9205 plates (Thermo Labsystems, USA) were coated 100 μl per well with SWA at 8 μg/ml or SEA at 2.4 μg/ml and incubated overnight at 4°C. The standard positive pool (prepared and optimised in-house at Department of Pathology, Cambridge University) was serially diluted and added in columns 1 and 2 of the plate and formed the standard curve for quantification of antibodies. Biotinylated mouse anti-human monoclonal antibodies used for the detection were obtained from BD Pharmingen (San Diego USA), except IgG3 which was obtained from Zymed (S. San Francisco, USA). Poly-HRP-streptavidin conjugate (Sanquin, Netherlands) was added at 1/4000 dilution. The plates were developed with OPD substrate and the reaction stopped by addition of 25 μl per well of 2 M sulphuric acid on observing the colour change. Optical densities (ODs) were recorded into a data file in DeltaSOFT II (BioMetalics, Inc USA) micro-plate analysis software programme and exported into Microsoft excel program. The ODs were interpolated into absolute concentration using Stata 5.0 (StataCorp, USA) generated standard curves. The sensitivity of the test was the lowest standard concentration above which levels were detectable.

### Statistical analysis

Data was entered in Microsoft Excel (Microsoft, USA) and imported into Stata (version 9 StataCorp, USA) for statistical analysis.

To assess the effect of pregnancy on the praziquantel-induced reduction in infection intensity, a simple table and χ^2 ^test was used to compare infection intensity categories at six weeks post treatment between women who were first treated during pregnancy (infection intensity at six weeks post-enrolment among praziquantel group) and those who were first treated at six weeks after delivery (infection intensity at 12 weeks after delivery among the initially placebo group).

Some antibody isotypes were detectable in less than 50% of the individuals, so antibody data was initially analysed as binary variables dichotomised according to whether the antibody was detectable or not. Subsequent analyses with the levels treated as quantitative variables were found to be more informative and are therefore presented, with results from both approaches consistent. Since the data was not normally distributed, non parametric statistical tests were used, except when comparing the boosts in antibody data that approached a normal distribution, when regression analysis was applied.

The analysis of schistosome antigen-specific antibodies had three main objectives:

1. To examine effect of pregnancy on antibody levels. Among the placebo group, antibody levels at enrolment and six weeks post-enrolment were compared with antibody levels at six weeks after delivery using Wilcoxon signed rank test.

2. To assess the effect of praziquantel treatment during pregnancy. First, within the group treated during pregnancy, antibody levels before and after praziquantel treatment were compared using Wilcoxon signed rank test. Then, levels at six weeks post-enrolment were compared between the placebo and praziquantel groups using Wilcoxon rank sum (Mann-Whitney) test.

3. To examine the influence of pregnancy on the praziquantel-induced boost in antibody levels at six weeks post-treatment. Initially the influence of infection intensity at enrolment and pre-treatment antibody levels on the boost in the antibody levels were explored using spearman's correlation analysis. The change (boost) in antibody levels during pregnancy among women first treated during pregnancy was compared with the change after delivery among women first treated after delivery using linear regression based on log_10_(antibody concentration+1).

## Results

**Figure 1:**
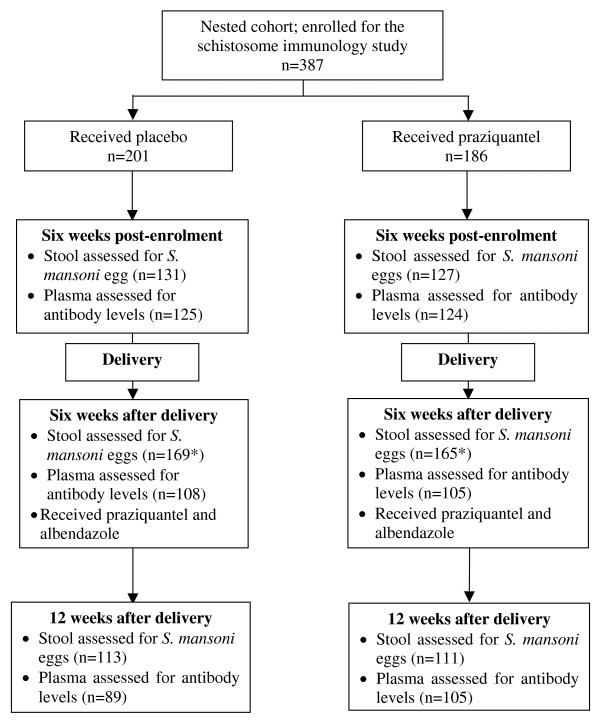
**Flow diagram showing the scheduled time points of the study and samples assessed at the respective time points**. *Women who missed attending or providing samples at some time points were allowed to participate at subsequent follow-up time points.

Between November 2003 and November 2005, 2208 women were recruited to the larger cohort of whom 387 (17.5%) had stool samples positive for *Schistosoma mansoni*; of these 186 women received praziquantel and 201 women received placebo. Due to some participants missing scheduled appointments or insufficient amounts of plasma samples to repeat failed antibody assays the numbers analysed for particular time points varied (figure 1). Characteristics of the women at enrolment, including intensity of schistosome infection, levels of antibodies against schistosome antigens and prevalence of co-infections such as hookworm, *P. falciparum *and HIV, were similar among the praziquantel and placebo groups (additional file 1). Except for *S. mansoni *infection intensity, none of these factors was associated with schistosome antigen-specific antibody levels at enrolment (data not shown). There were positive correlations between *S. mansoni *infection intensity and levels of IgG1 (p < 0.001), IgG2 (p = 0.01), IgG4 (p < 0.001) and IgE (p < 0.001) against SWA and IgG4 against SEA while there were negative correlations between S. mansoni infection intensity and levels of IgG1 (p = 0.005) IgG2 (p < 0.001) and IgG3 (p = 0.005) against SEA.

### The effect of praziquantel on *S. mansoni *infection intensity was similar when given during pregnancy or after delivery

Among women randomised to praziquantel during pregnancy, 104/127 (81.9%) were negative for *Schistosoma mansoni *at six weeks post-treatment while 11.8%, 4.7% and 1.6% still had light, moderate and heavy infection respectively. In the placebo group who were later treated after delivery, 100/113 (88.5%) were negative while 10.6% and 0.9% still had light and moderate infection respectively at six weeks post-treatment, while none had heavy infection. Thus although the residual intensity was slightly higher in the group treated during pregnancy, this effect was small (p = 0.16). Further, there was no evidence of an effect of gestational age at the time of treatment among those treated during pregnancy (data not shown). Albendazole did not influence the effects of praziquantel treatment against *S. mansoni *nor did praziquantel influence the effects of albendazole against hookworm (data not shown).

### Levels of antibodies against SWA and SEA are reduced during pregnancy

**Figure 2:**
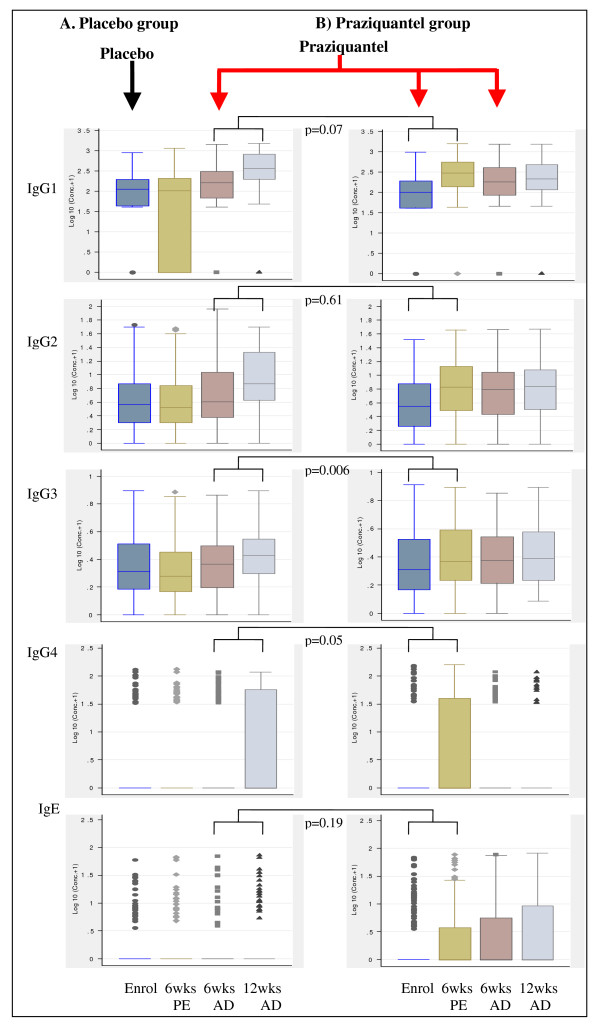
**Plasma levels of antibodies against *S. mansoni *adult worm antigen (SWA) and effect of praziquantel treatment during pregnancy or after delivery on the antibody levels**. The y-axis shows log_10 _(antibody concentrations (pg/ml)+1). The x-axis shows time points: enrolment (Enrol), 6 weeks post-enrolment (6 wks PE), 6 weeks after delivery (6 wksAD) and 12 weeks after delivery (12 wks AD). The arrows indicate the intervention time points when the women were given either placebo (black) or praziquantel treatment (red). The box plots in column A are for participants who received placebo at enrolment during pregnancy and received praziquantel treatment for the first time six weeks after delivery. The box plots in column B are for participants who received praziquantel treatment during pregnancy. P values are given for the comparison in boost in antibody levels for praziquantel given for the first time during pregnancy vs after delivery, adjusted for pre-treatment antibody levels, *S. mansoni *infection intensity at enrolment and concurrent albendazole therapy. P values for other comparisons are given in the text.

**Figure 3:**
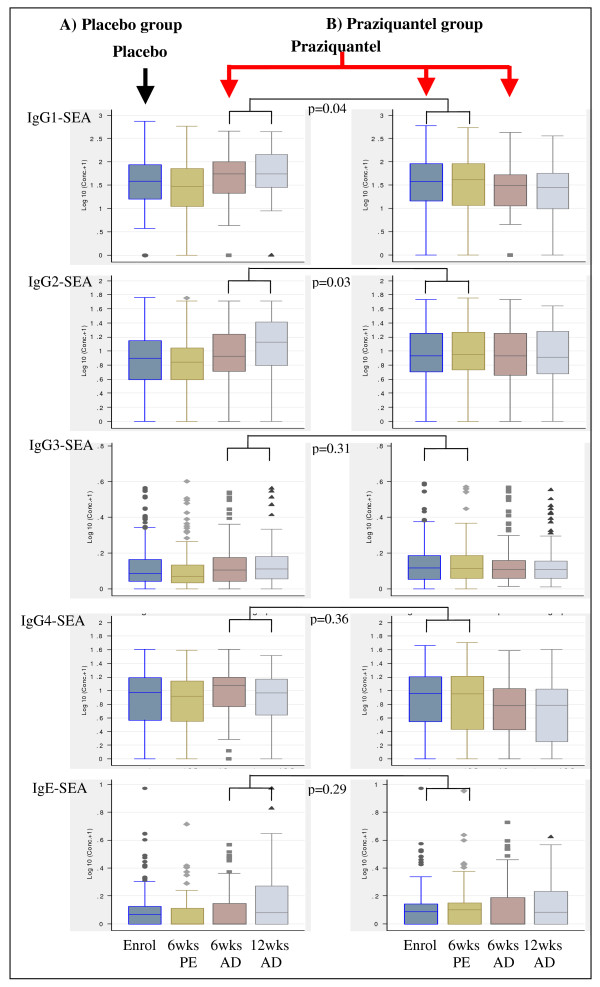
**Plasma levels of antibodies against *S. mansoni *egg antigen (SEA) and effect of praziquantel treatment during pregnancy or after delivery on the antibody levels**. The y-axis shows log_10 _(antibody concentrations (pg/ml)+1). The x-axis shows time points: enrolment (Enrol), 6 weeks post-enrolment (6 wks PE), 6 weeks after delivery (6 wksAD) and 12 weeks after delivery (12 wks AD). The arrows indicate the intervention time points when the women were given either placebo (black) or praziquantel treatment (red). The box plots in column A are for participants who received placebo at enrolment during pregnancy and received praziquantel treatment for the first time six weeks after delivery. The box plots in column B are for participants who received praziquantel treatment during pregnancy. P values are given for the comparison in boost in antibody levels for praziquantel given for the first time during pregnancy vs after delivery, adjusted for pre-treatment antibody levels, *S. mansoni *infection intensity at enrolment and concurrent albendazole therapy. P values for other comparisons are given in the text.

Among the placebo group, levels of antibodies against SWA (figure 2A) and SEA (figures 3A), at enrolment and six weeks post-enrolment during pregnancy were consistently lower than at six weeks after delivery; and this effect was strong at enrolment for IgG1 (p < 0.001), IgG2 (p = 0.018), IgG4 (p = 0.002) and IgE (p < 0.001) against SWA, and for IgG1 (p = 0.014) and IgG4 (p = 0.032) against SEA and at six weeks post-enrolment for IgG1 (p < 0.001), IgG2 (p = 0.005), IgG3 (p = 0.012), IgG4 (p = 0.001) and IgE (p = 0.018) against SWA, and IgG1 (p = 0.001), IgG3 (p = 0.009) and IgG4 (p = 0.008) against SEA.

### Praziquantel treatment during pregnancy causes boosts in levels of antibodies against SWA as well as IgG3 and IgE against SEA

Among the women who received praziquantel at enrolment, there was a significant boost in levels of IgG1 (p < 0.001), IgG2 (p < 0.001), IgG3 (p = 0.011) and IgG4 (p < 0.001) against SWA at six weeks post-treatment (figure 2B). When antibody levels at six weeks post-enrolment were compared between the placebo and the praziquantel groups, the levels of antibodies against SWA were higher in the praziquantel group than in the placebo group (p < 0.001 for IgG1-3, p = 0.034 for IgG4, p = 0.049 for IgE and p = 0.013 for IgM). Levels of IgE against SWA did not show significant boost at six weeks post-treatment, but at six weeks after delivery IgE levels were significantly higher than pre-treatment (p < 0.001) and six weeks post-treatment (p = 0.006), and the level among women treated during pregnancy was again higher than among those who received placebo (p = 0.018). IgG subtypes and IgM showed no further increase after delivery.

In contrast, boosts in antibodies against SEA were only seen for IgG3 (p-value= 0.02) and IgE (p-value = 0.043), with levels at six weeks post-treatment higher than pre-treatment levels (figure 3B). When antibody levels at six weeks post-enrolment were compared between the placebo and praziquantel groups, levels of IgG2 (p = 0.006), IgG3 and IgE (p < 0.001) against SEA were higher among the praziquantel group than the placebo group. On the other hand, the level of IgG4 against SEA showed significant decline between enrolment and six weeks post-treatment (p = 0.02) and at six weeks post-enrolment the levels were not significantly different between the placebo and praziquantel group.

### Pregnancy causes suppression of praziquantel induced-boost in levels of some antibodies against SWA and SEA

Antibody levels in the group randomised to placebo and who received initial praziquantel treatment at six weeks after delivery were examined at 6 weeks post-treatment to assess the effect of treating non-pregnant women (figures 2A and 3A). Significant boosts in antibodies against SWA (p < 0.001 for IgG1-4, and IgM) as well as IgG2 (p < 0.001) and IgE (p = 0.006) against SEA were observed at six weeks after post-delivery treatment. As observed with treatment during pregnancy, levels of IgG4 against SEA (p = 0.007) significantly declined following post-delivery praziquantel treatment.

Comparing post-treatment antibody levels by timing of treatment showed that the boosts in antibody levels were generally lower for treatment during pregnancy than for treatment after delivery (additional file 2). As well as absolute antibody levels, boosts in antibody levels against SWA tended to show a positive association with infection intensity at enrolment (data not shown). After adjusting for the pre-treatment antibody levels, infection intensity at enrolment and albendazole treatment, the post-treatment boosts remained lower for the women first treated during pregnancy than those first treated after delivery, particularly significant for IgG3, IgG4 and IgM against SWA and for IgG1 and IgG2 against SEA (additional file 2).

## Discussion

This study suggests that praziquantel treatment against *S. mansoni *during pregnancy has similar efficacy as praziquantel treatment in non-pregnant women, despite the presence of lower levels of anti-schistosome antibodies and tendency to reduced boosting of both antibody and cytokine responses observed during pregnancy.

In assessing effects on *S. mansoni *infection intensity, the chief limitation of this study was the use of a single stool sample to determine prevalence and intensity of *S. mansoni *after treatment. A single stool sample has low sensitivity in detection of helminth infections [29-31], hence the prevalence will have been underestimated and estimates of intensity will have been imprecise. However, since comparisons were made between randomised groups, these limitations are likely to have applied similarly at follow up in both groups; the proportion apparently "cured" will have been exaggerated in both groups, but the comparison remains valid, suggesting little difference in the effect of treatment during pregnancy or after delivery.

In this study, alterations in the levels of schistosome antigen-specific antibodies during pregnancy, as well as the effect of praziquantel treatment during pregnancy on the antibody levels, were elucidated. Levels of IgG1, IgG2, IgG3, IgG4 and IgE against SWA and SEA, were generally low during pregnancy. These findings accord those of Novato-Silva and colleagues [32] showing that antibody levels against adult schistosome worm antigens declined with progression of pregnancy. These observations are contrary to other reports on antibodies to non-schistosome antigens showing that humoral immune responses are not affected by progression of pregnancy [19,33]. There is normally an increase in plasma and blood volume during pregnancy [34] which is apparent at six weeks, and is maximum (40–50%) between 32–36 weeks, of pregnancy [34,35]. Although this dilution effect of increased blood volume of pregnancy was not examined in our study, it could potentially explain the low antibody levels during pregnancy rather than a decline in the antibody responses *per se*.

Praziquantel treatment during pregnancy caused a boost in antibody levels against SWA and to a limited extent SEA; although a significant drop in levels of IgG4 against SEA was observed. These boosts in antibodies at six weeks post-treatment were consistent with those observed at five weeks post-treatment [36], three months post-treatment [37] and at six months post-treatment [27] among *S. mansoni *infected non-pregnant individuals in endemic areas. Similar increases in antibodies were recently reported following treatment among non-pregnant adults from a new focus of *S. mansoni *infection [38]. The decline in the level of IgG4 against SEA following praziquantel treatment was also consistent with findings reported among non-pregnant individuals [37,39]. The boost in IgE against SWA was less marked than for other antibodies against SWA. It is possible that six weeks after treatment could have been too short a follow-up time to observe a significant boost in levels of IgE against SWA. This was consistent with other studies where post-treatment levels of IgE against SWA were not significantly boosted at three weeks [26] and five weeks [36], following treatment, suggesting that this particular isotype remains relatively stable over time among adults in *S. mansoni *endemic areas.

Although boosts in schistosome antigen-specific antibody response were observed during pregnancy, these were generally lower, and post-treatment antibody levels were lower, compared with treatment after delivery. However, in spite of this, the data did not show evidence that pregnancy and having lower boosts in schistosome antigen specific antibody isotypes had any significant impact on the efficacy of praziquantel treatment against *S. mansoni*. Suppression of the boosts in antibodies during pregnancy may, however, have effects on resistance to reinfection. Due to limited follow-up time and since all the women received praziquantel treatment after delivery, the impact of treatment during pregnancy on susceptibility to reinfection after treatment was not explored. Therefore, further studies might be necessary to examine the long-term implications of the suppression of boosts in some of the antibody isotypes following treatment during pregnancy.

## Conclusion

This study, the first to examine the impact of praziquantel treatment during pregnancy, has shown that *S mansoni *antigen-specific antibody levels and praziquantel-induced boosts in the antibody levels are broadly low during pregnancy, but that this is not associated with any major reduction in the efficacy of praziquantel in the treatment of light to moderate intensity *S. mansoni *infections: a result that is reassuring from a public health perspective. Effects of pregnancy on the response to praziquantel among populations with higher *S. mansoni *infection intensities, and among women with *S. haematobium *or *S. japonicum*, and the long-term implications of these findings, particularly in relation to resistance to re-infection, remain to be explored.

## Competing interests

The authors declare that they have no competing interests.

## Authors' contributions

RT participated in designing the study, carried out the immunoassays, did the data analysis, drafted the manuscript and coordinated writing the manuscript. PMA participated in the immunoassays. NOE participated in the parasitological analysis of stool samples. HP: participated in collection of the samples and coordination of participants. FMJ participated in the immunoassays. TD participated in the data analysis. DWD participated in designing the study and writing the manuscript. BJV participated in designing the study and writing the manuscript. EKR contributed in conducting the study. AME conceived the idea of the study, participated in designing the study, data analysis and coordinated writing of the manuscript. All the authors read and approved the final manuscript.

## Pre-publication history

The pre-publication history for this paper can be accessed here:

http://www.biomedcentral.com/1471-2334/9/32/prepub

## Supplementary Material

Additional file 1 **Table 1.** Comparison of characteristics at enrolment between the praziquantel and placebo groups Click here for file

Additional file 2 **Table 2.** Comparison of the boost in levels of antibodies against SWA and SEA at six weeks following praziquantel treatment during pregnancy or after delivery Click here for file
