# Supplementary material for: Effect of praziquantel treatment of Schistosoma mansoni during pregnancy on intensity of infection and antibody responses to schistosome antigens: results of a randomised, placebo-controlled trial
Source: BMC Infect Dis. 2009 Mar 18;9:32. doi: 10.1186/1471-2334-9-32 (PMC2666740; doi:10.1186/1471-2334-9-32)
Supplement: Additional file 1 — Table 1. Comparison of characteristics at enrolment between the praziquantel and placebo groups [file 1471-2334-9-32-S1.doc]

**Table 1. Comparison of characteristics at enrolment** between the praziquantel and placebo groups

|  | |  | **Placebo group**  **n=201** | **Praziquantel group n=186** |
| --- | --- | --- | --- | --- |
| **Characteristics of the women:** | |  |  |  |
| Median age in years (interquartile range) | |  | 22 (19, 26) | 22 (19, 26) |
| Primigravida (number (%)) | |  | 44 (21.9%) | 58 (31.2%) |
| Median gestational age in days at time of treatment (IQR) | |  | 190(158, 217) | 186 (153, 216) |
| ***S. mansoni* infection intensity:** | |  |  |  |
|  | Light  Moderate  Heavy | | 133 (66.2%)  39 (19.4%)  29 (14.4%) | 119 (64.0%)  36 (19.3%)  31 (16.7%) |
| **Hookworm infection (number (%))** | |  | 76 (37.8%) | 81 (43.5%) |
| **Number (%) who received albendazole** | |  | 104 (51.7%) | 97 (52.1%) |
| ***P. falciparum* infection (proportion (%) positive)** | |  | 18/197 (9.9%) | 21/183 (11.5%) |
| **HIV infection (number (%) positive)** | |  | 26 (12.9%) | 22 (11.8%) |
| **Antibodies to SWA:** (number (%) with detectable levels and median (inter-quartile range) (µg/ml) | |  | | |
|  |  | IgG1 | 155 (77.9%)  108.4 (42.6, 192.7) | 141 (75.8%)  99.3 (40.1, 186.0) |
|  |  | IgG2 | 163 (81.9%)  2.6 (1.0, 6.4) | 143 (76.9%)  2.5 (0.8, 6.5) |
|  |  | IgG3 | 187 (94.0%)  1.1 (0.5, 2.3) | 177 (95.1%)  1.0 (0.5, 2.3) |
|  |  | IgG4 | 30 (15.1%)  0 (0, 0) | 27 (14.5%)  0 (0, 0) |
|  |  | IgE | 29 (14.6%)  0 (0, 0) | 38 (20.1%)  0 (0, 0) |
|  |  | IgM | 199 (100%)  11.7 (7.4, 18.9) | 186 (100%)  11.9 (8.2, 18.3) |
| **Antibodies to SEA:** number (%) with detectable levels and median (inter-quartile range) (µg/ml) | |  | |  |
|  |  | IgG1 | 181 (90.9%)  37.7 (15.4, 86.8) | 163 (87.6%)  36.3 (13.6, 90.1) |
|  |  | IgG2 | 193 (97.0%)  6.9 (3.0, 13.1) | 178 (96.2%)  7.6 (4.1, 17.1) |
|  |  | IgG3 | 189 (95.0%)  0.2 (0.1, 0.5) | 179 (96.2%)  0.3 (0.1, 0.5) |
|  |  | IgG4 | 175 (87.9%)  8.4 (2.7, 14.4) | 161 (86.6%)  8.2 (2.5, 15.0) |
|  |  | IgE | 104 (52.2%) | 104 (55.9%) |
|  |  |  | 0.2 (0, 0.3) | 0.2 (0, 0.4) |
