# Supplementary material for: Effect of praziquantel treatment of Schistosoma mansoni during pregnancy on intensity of infection and antibody responses to schistosome antigens: results of a randomised, placebo-controlled trial
Source: BMC Infect Dis. 2009 Mar 18;9:32. doi: 10.1186/1471-2334-9-32 (PMC2666740; doi:10.1186/1471-2334-9-32)
Supplement: Additional file 2 — Table 2. Comparison of the boost in levels of antibodies against SWA and SEA at six weeks following praziquantel treatment during pregnancy or after delivery [file 1471-2334-9-32-S2.doc]

**Table 2 Comparison of the boost in levels of antibodies against SWA and SEA at six weeks following praziquantel treatment during pregnancy or after delivery**

|  | median change in antibody levels , µg/ml (inter-quartile range) | |  | Difference in the change in log_10_ (antibody concentration +1) (95% confidence interval) for treatment in pregnancy versus after delivery* | |
| --- | --- | --- | --- | --- | --- |
| Antigen-antibody | First treated during pregnancy | First treated after delivery |  | crude analysis | adjusted analysis^†^ |
| SWA |  |  | | | |
| IgG1 | 159.2 (62.3, 359.6) | 204.4 (78.2, 435.7) |  | -0.03 (-0.21, 0.14) | -0.15 (-0.31, 0.01), p=0.07 |
| IgG2 | 2.1 (0, 6.0) | 2.3 (0.3, 7.6) |  | -0.002(-0.1, 0.10) | -0.03 (-0.1, 0.08), p=0.61 |
| IgG3 | 0.06 (-0.1, 0.5) | 0.2 (0, 0.7) |  | -0.04 (-0.08, -0.01) | -0.04 (-0.08, -0.01), p=0.006 |
| IgG4 | 0 (0, 0) | 0 (0, 24.7) |  | -0.1 (-0.3, 0) | -0.18 (-0.3, 0), p=0.05 |
| IgE | 0 (0, 0) | 0 (0, 0) |  | -0.05 (-0.15, 0.04) | -0.07 (-0.19, 0.03), p=0.19 |
| IgM | 1.0 (-2.2, 3.2) | 1.8 (-2.2, 5.8) |  | -0.04 (-0.07, 0) | -0.07 (-0.09, -0.01), p=0.01 |
| SEA |  |  | | | |
| IgG1 | -1.6 (-21.6, 17.2) | 2.6 (-20.2, 40.2) |  | -0.04 (-0.21, 0.12) | -0.18 (-0.36, -0.01), p=0.04 |
| IgG2 | 0.1 (-2.4, 2.8) | 1.0(-0.8, 7.8) |  | -0.08 (-0.14, -0.02) | -0.08 (-0.15, -0.01), p=0.03 |
| IgG3 | -0.01 (-0.11, 0.1) | 0 (-0.05, 0.1) |  | -0.01 (-0.02, 0) | -0.01 (-0.02, 0.01), p=0.31 |
| IgG4 | -1.0 (-4.1, 0.3) | -0.7 (-3.1, 0.9) |  | -0.02 (-0.08, 0.03) | -0.03 (-0.09, 0.03), p=0.36 |
| IgE | 0 (-0.02, 0.20) | 0 (0, 0.3) |  | -0.01 (-0.05, 0.02) | -0.02 (-0.06, 0.01), p=0.29 |

* Shown are the regression coefficients which are the differences of the mean change in log_10_ (antibody concentration +1) at six weeks following treatment during pregnancy compared to that at six weeks following initial treatment after delivery. A negative sign implies that the boost in antibody levels following treatment during pregnancy was lower than the boost following treatment after delivery.

^†^ adjusted for pre-treatment antibody levels, *S. mansoni* infection intensity at enrolment and concurrent albendazole therapy
